# The Association between Plasma Levels of Trimethylamine N-Oxide and the Risk of Coronary Heart Disease in Chinese Patients with or without Type 2 Diabetes Mellitus

**DOI:** 10.1155/2018/1578320

**Published:** 2018-08-08

**Authors:** Zengxiang Dong, Zhaoguang Liang, Meihua Guo, Shuang Hu, Zhaoqian Shen, Xin Hai

**Affiliations:** ^1^Department of Pharmacy, The First Affiliated Hospital of Harbin Medical University, Harbin 150001, China; ^2^Department of Cardiology, The First Affiliated Hospital of Harbin Medical University, Harbin 150001, China

## Abstract

**Aim:**

Trimethylamine N-oxide (TMAO) has been demonstrated as an independent risk factor for cardiovascular disease. Our objective was to determine the plasma levels of TMAO in Chinese coronary heart disease (CHD) patients with or without type 2 diabetes mellitus (T2DM).

**Methods:**

A total of 132 control participants, 243 CHD patients, and 175 CHD patients with T2DM were enrolled. Plasma levels of TMAO in all patients were measured and analyzed.

**Results:**

The plasma levels of TMAO were significantly higher in CHD patients than in control subjects (3.08 ± 0.13 *μ*M versus 1.49 ± 0.05 *μ*M; *P* < 0.01). In addition, plasma levels of TMAO were remarkably increased in CHD patients with T2DM compared with CHD patients (7.63 ± 0.97 *μ*M versus 3.08 ± 0.13 *μ*M; *P* < 0.01). The receiver operating characteristic analysis revealed that the area under the curve of TMAO was 0.794 and 0.927 to predict CHD or CHD-T2DM patients (*P* < 0.01). Univariate and multivariate logistic regression analysis showed that TMAO was an independent predictor in CHD patients with or without T2DM. The level of TMAO was correlated with high-sensitive troponin I (hs-TnI) and creatine kinase MB (CKMB).

**Conclusions:**

TMAO was an independent predictor of CHD in Chinese patients; moreover, the TMAO levels were highly associated with diabetes in CHD patients.

## 1. Introduction

Coronary heart disease (CHD) is a major cause of morbidity and mortality in people with diabetes mellitus. Type 2 diabetes mellitus (T2DM) is a metabolic disorder characterized by systemic insulin resistance, which promotes hyperglycemia [1], and it has been found that these metabolic abnormalities lead to increased risk of cardiovascular diseases. So far, the occurrence and development of CHD in diabetic patients are complex and not fully understood [2]. The clinical importance of identifying CHD-T2DM risk is that it can contribute to the determination of the treatment and prevention method of the disease. When both CHD and T2DM occur, a comprehensive prevention of cardiovascular risk factors should be performed in order to reduce the morbidity and mortality of the disease. Rather than simply lowering blood glucose or blood pressure, the comprehensive treatment strategy should include correcting abnormity of blood lipid, glucose, and pressure, reasonable exercise, weight control, and cessation of smoking. In particular, many studies have indicated that diet pattern plays an important role in CHD-T2DM risk [3, 4].

Trimethylamine N-oxide (TMAO) is a plasma metabolite from nutrient precursors (choline, phosphatidylcholine, and L-carnitine), which is produced by gut microbiota. Many studies indicated the participation of gut microbes in the pathogenesis of cardiovascular disease [5–8]. Furthermore, numerous studies have revealed a relation between plasma levels of TMAO and cardiovascular risks [9–17]. These studies have indicated that elevated plasma levels of TMAO were each associated with incident risk of major adverse cardiac events independent of traditional risk factors [9], some of which found that high TMAO levels portended higher long-term adverse clinical outcomes in patients with heart failure [10, 11], and plasma levels of TMAO were an independent predictor of a high atherosclerotic burden in patients with CHD [15]. Moreover, a study has found that diabetes was associated with higher TMAO plasma levels [14]. However, the further relationship between TMAO levels and risks for CHD in patients with T2DM has not yet been examined. A rational hypothesis is thus that circulating levels of TMAO could predict and induce incident risks for CHD among subjects presenting with T2DM, which could provide new idea and method for the prevention of CHD-T2DM. Herein, we sought to explore the relationship between plasma levels of TMAO and CHD among patients with T2DM.

## 2. Methods

### 2.1. Participants

Between May 2016 and August 2017, 550 patients presented to the First Affiliated Hospital of the Harbin Medical University (Harbin, China). The study consisted of three subject groups: control subjects (*n* = 132), CHD patients (*n* = 243), and CHD patients with T2DM (*n* = 175). The clinical characteristics of the study population are summarized in Table 1.

### 2.2. Ethical Approval of Studies and Informed Consent

The study protocols and the procedures for handling human samples were approved by the Ethics Committee of the First Affiliated Hospital of Harbin Medical University (Harbin, China), and all patients gave informed consent. All the methods were carried out in accordance with the approved guidelines.

### 2.3. Collection and Handling of Human Blood Samples

Whole blood samples (1 mL per patient) were drawn from the study subjects via a direct venous puncture into the tubes containing EDTA. The human whole blood samples in vacuum tubes were kept at 4°C and then centrifuged at 765*g* (centrifuge: Allegra 64R, Beckman Coulter, USA; rotor: Beckman F1010, USA) for 15 min at 4°C to obtain plasma samples.

### 2.4. Determination of TMAO by LC-MS/MS

We measured TMAO by LC-MS/MS using 50 *μ*M TMAO-d9 (TRC, Canada) as an internal standard according to the previous method [17]. Plasma was deproteinized by a mixture with an internal standard solution and methanol (19 : 1 : 60 vol : vol : vol). 5 *μ*L of each sample supernatant was injected in an Agilent 1100 high-performance liquid chromatography system (Agilent Technologies, USA), and analytes were separated on a Phenomenex Luna Silica column (100 mm × 2 mm, 3 *μ*m particle size) protected by a guard column (4 mm × 2 mm silica filter) at room temperature. The mobile phase consisted of 88% methanol containing 10 mM ammonium formate and 0.2% formic acid (*v*/*v*) and 12% of 10 mM ammonium formate in water containing 0.2% formic acid (*v*/*v*) at a flow rate of 0.2 mL/min. MS was performed on an API 4000 triple quadrupole mass spectrometer (AB Sciex, USA) with electrospray ionization in the positive mode. Ion transitions used for quantitation were *m*/*z* 76 → 58 for TMAO and *m*/*z* 85 → 66 for the internal standard.

### 2.5. Statistical Analysis

Continuous variables were described as mean ± SD (standard deviation). Categorical variables were presented as the percentage of total patients. Differences between groups in continuous variables and in categorical variables were analyzed by the unpaired 2-tailed *t*-test and by the chi-squared test, respectively. Univariate and multivariate logistic regression analyses were used to assess the association between the risk of TMAO and CHD/CHD-T2DM patients. Odds ratios (OR) were presented with 95% confidence intervals (CI) to show the risk of an event when a factor was apparent. To evaluate the levels of TMAO to predict CHD/CHD-T2DM patients, the receiver operating characteristic (ROC) curves were constructed and the area under the curves (AUC) were calculated. The correlation coefficient for the association of TMAO with conventional prognostic markers of CHD patients was measured by Pearson's correlation analysis. All analyses were carried out with SPSS v17.0 software. Statistical significance was accepted at *P* < 0.05 for all analyses.

## 3. Result

### 3.1. Clinical Characteristics of the Study Population

The study consisted of three subject groups: 132 control subjects, 243 CHD patients, and 175 CHD patients with T2DM. Table 1 shows the clinical and demographic characteristics of the patients enrolled in this study. In all the subject groups, there was no statistically significant difference in the age and sex distribution (*P* > 0.05), whereas high-sensitive troponin I (hs-TnI) and creatine kinase MB (CKMB) levels differed significantly between CHD patients with or without T2DM and control (*P* < 0.05).

### 3.2. Plasma Levels of TMAO in CHD and CHD-T2DM Patients

**Figure 1:**
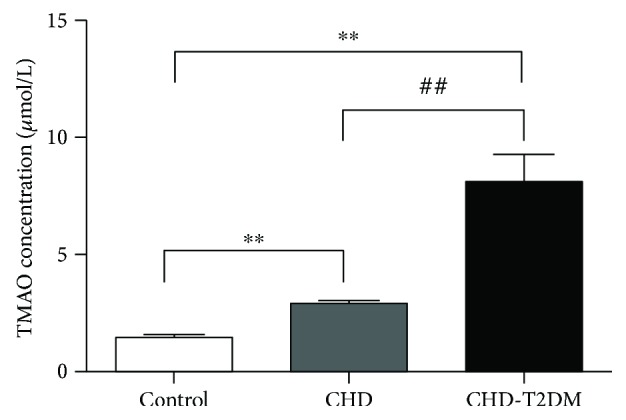
The plasma levels of TMAO. TMAO levels were confirmed in control, CHD, and CHD-T2DM, respectively. ^∗∗^*P* < 0.01 versus control group, ^##^*P* < 0.01 versus CHD group.

LC-MS/MS analysis was used to determine the concentration of TMAO in plasma. As illustrated in Figure 1, TMAO demonstrated significant differences in plasma samples between control, CHD, and CHD-T2DM. The levels of circulating TMAO were significantly higher in CHD patients than in control subjects (3.08 ± 0.13 *μ*M versus 1.49 ± 0.05 *μ*M; *P* < 0.01). In addition, plasma levels of TMAO were remarkably increased in CHD patients with T2DM compared with control (7.63 ± 0.97 *μ*M versus 1.49 ± 0.05 *μ*M; *P* < 0.01) or CHD patients (7.63 ± 0.97 *μ*M versus 3.08 ± 0.13 *μ*M; *P* < 0.01).

### 3.3. Evaluation of TMAO as a New Biomarker for CHD and CHD-T2DM

**Figure 2:**
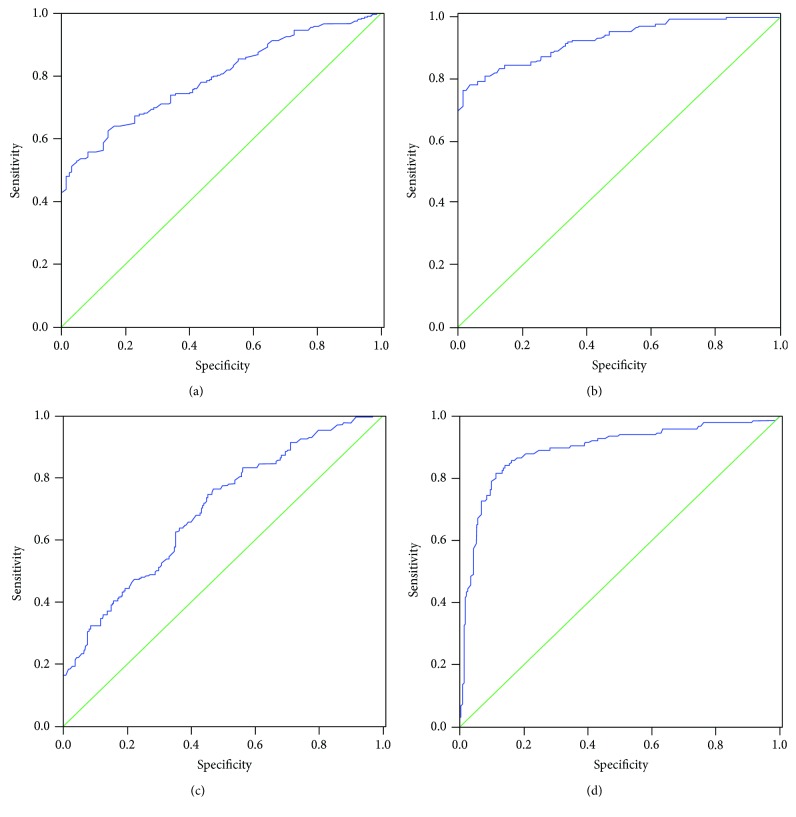
Receiver operator characteristic analysis of TMAO for predicting CHD and CHD-T2DM. The area under ROC curve was determined to evaluate the predictive power of circulating TMAO levels for CHD (a) and CHD-T2DM (b). The area under ROC curve was determined to evaluate the predictive power of circulating TMAO levels (c) and blood glucose levels (d) for CHD-T2DM.

According to the established fact that TMAO present in plasma levels were significantly altered in CHD/CHD-T2DM patients, we sought to determine the potential utility of circulating TMAO as a diagnostic biomarker of CHD and CHD-T2DM. For this purpose, ROC analysis was performed to evaluate the predictive power of circulating TMAO for control, CHD, and CHD-T2DM. Our results showed that the area under ROC curve of TMAO was 0.794 (95% CI = 0.751~0.838) for CHD and 0.927 (95% CI = 0.899~0.954) for CHD-T2DM (Figures 2(a) and 2(b)). Meantime, we used the area under ROC curve to evaluate the predictive power of circulating TMAO levels and blood glucose levels for CHD-T2DM. Our results showed that the area under ROC curve of TMAO was 0.697 (95% CI = 0.646~0.747) for CHD-T2DM and the area under ROC curve of blood glucose was 0.894 (95% CI = 0.859~0.930) for CHD-T2DM (Figures 2(c) and 2(d)).

The univariate analysis with logistic regression showed that the odds ratio (OR) value was 3.621 (95% CI: 2.581~5.080) for TMAO (*P* < 0.01) between control and CHD and the OR value was 9.581 (95% CI: 5.451~16.840) for TMAO (*P* < 0.01) between control and CHD-T2DM (Table 2). The multivariate logistic regression analysis further verified TMAO as an independent predictor for CHD-T2DM: the OR value was 3.469 (95% CI: 2.294~5.245, *P* < 0.01) for TMAO between control and CHD and the OR value was 9.110 (95% CI: 4.427~18.750, *P* < 0.01) for TMAO between control and CHD-T2DM (Table 3).

### 3.4. Relation of TMAO to Conventional Prognostic Markers of CHD

The risk of CHD can be estimated by determining the hs-TnI and CKMB. To further evaluate the usefulness of circulating TMAO as a CHD or CHD-T2DM biomarker, we tested whether the level of TMAO was correlated with conventional prognostic markers of CHD. The data summarized in Table 4 shows that TMAO is positively correlated with hs-TnI and CKMB in CHD and CHD-T2DM.

## 4. Discussion

The present study provides new information about the relation between plasma levels of TMAO and CHD/CHD-T2DM in Chinese patients. It had been reported that TMAO was a significant risk factor of cardiovascular disease, which had been demonstrated as an independent predictor of a high atherosclerotic burden and heart failure in patients [10, 15]. Although these previous studies consisted of large numbers of patients, there was no study focusing on the relation between TMAO and Chinese CHD/CHD-T2DM patients. We demonstrated that the TMAO was an independent predictor of CHD beyond the traditional prognostic markers in CHD patients. The further information of TMAO could provide an incremental predictive value for the risk of CHD-T2DM, over the traditional prognostic markers, which was based on statistical analysis of clinical variables.

Recently, CHD still remains the leading cause of death worldwide [18–20] and is also a major cause of morbidity and mortality in people with T2DM [3]. In order to improve outcomes of patients with cardiovascular disease, many studies have focused on novel modifiable risk factors, with a special attention to environmental sources [21, 22]. Although diet has always been linked to outcomes of cardiovascular disease, the important role of gut microbiota has only recently been recognized in the field of cardiovascular disease. Hence, we have sought to research the impact of the gut microbiota-dependent metabolite, TMAO, on CHD in Chinese patients with or without T2DM. Our study demonstrated that circulating TMAO levels were elevated in both patients with CHD and CHD-T2DM. Another novel finding in our results is that circulating TMAO was able to discriminate T2DM patients with CHD from CHD patients and control subjects with an area under the curve of the ROC of 0.927 (95% CI = 0.899~0.954) and 0.794 (95% CI = 0.751~0.838), respectively, which suggested that circulating TMAO exhibited not only a potential biomarker for CHD but also a biomarker for predicting CHD patients with T2DM. Our results were further confirmed in univariate regression analysis and multivariate logistic regression analysis which revealed strong association of TMAO expression with CHD/CHD-T2DM. We also showed that circulating TMAO was significantly and positively correlated with hs-TnI and CKMB in CHD/CHD-T2DM, as determined using Pearson's correlation analysis.

A recent study suggests that plasma levels of TMAO among patients with chest pain may be a risk predictor of incident cardiovascular events and may provide clinical benefit in risk stratification among patients with acute coronary syndromes [23]. Meantime, a clinical study exploring the prognostic value of TMAO in patients indicated that the strength of the association between TMAO and adverse events differed somewhat by race, with whites showing a significant association between elevated TMAO levels and heightened incident risk of cardiac death and any-cause death, whereas in blacks, higher TMAO levels were only significantly associated with cardiac death [24]. Our results in Chinese are consistent with these other clinical studies in that they show elevated levels of TMAO in cardiovascular disease. However, to determine if TMAO may serve as an independent risk factor for long-term mortality, further study is needed in the future.

TMAO induced cardiovascular disease via a multiplicity of effects, including changes in macrophage and endothelial dysfunction induced by vascular oxidative stress and inflammation [25–27]. In addition, TMAO has an effect on platelet activation, inducing platelet hyperreactivity leading to a prothrombotic effect and promoting thrombus formation [8]. All these effects are important mechanisms of TMAO-induced CHD. In our study, we found that the levels of TMAO were remarkably increased in CHD patients with T2DM compared with CHD patients. The result was consistent with that of the previous study [14, 28]. TMAO has also been suggested to be a strong candidate molecule to mediate the development of T2DM in animal and human studies [29]. On the other hand, T2DM patients presented with an alteration in gut microbiota equilibrium, a disruption of gut barrier function, and an increase in gut permeability which altogether might result in aberrant production and absorption of TMAO [28]. The high levels of TMAO induced by T2DM could be influencing the progression of CHD through alterations in cholesterol and bile acid metabolism, activation of inflammatory pathways, and promotion of foam cell formation. This situation will increase the risk of major adverse cardiovascular events and death in the CHD-T2DM patients.

Furthermore, the previous study result suggested that decreasing the plasma levels of TMAO by selectively inhibiting microbial trimethylamine (TMA) production with a drug could serve as a means of inhibiting diet-induced atherosclerosis in animal models [30]. Similarly, the suppression of TMAO with approaches to knock down flavin monooxygenase 3 (the major hepatic enzyme responsible for the conversion of microbial-generated TMA into TMAO) could also inhibit atherosclerosis in animal models [31–33]. Thus, these results indicated that TMAO not only appeared to be a risk marker but also might serve as a potential therapeutic target to treat disease, including patients with CHD or CHD-T2DM. Therefore, we speculate that rapid and accurate determination of TMAO levels could significantly improve rapid diagnosis and risk stratification between patients presenting with CHD and CHD-T2DM, which could improve the effect of treatment on the disease. At present, TMAO is known to be a plasma biomarker in patients at high risk for the development of T2DM and advanced chronic kidney disease [34]. In our study, we suggest that circulating TMAO may be a risk factor in T2DM-induced CHD, but the study points to TMAO as a potential therapeutic target for the clinical management of T2DM-induced CHD, which needs further study.

## 5. Conclusions

TMAO was an independent predictor of CHD; moreover, the TMAO levels were highly associated with diabetes in Chinese CHD patients, which could potentially refine CHD stratification in diabetes patients. The importance of our findings is underlined by the modifiable nature of TMAO, both with diet and with potential therapeutics under development, offering new prospects in treatment strategies for Chinese CHD patients with or without T2DM.

## Figures and Tables

**Table 1:** Baseline characteristics of the patients.

Characteristic	Control (*n* = 132)	CHD (*n* = 243)	CHD-T2DM (*n* = 175)
Age (years)	61.9 ± 14.8	61.8 ± 11.1	64.4 ± 10.9
Male (%)	48 (36.4%)	109 (44.9%)	76 (43.4%)
Hypertension (%)	37 (28.0%)	129 (53.1%)^∗^	118 (67.4%)^∗^^#^
Current smoking (%)	3 (2.3%)	20 (8.2%)^∗^	19 (10.9%)^∗^^#^
Alcohol intake (%)	42 (31.8%)	67 (27.6%)	59 (33.7%)
HDL (mmol/L)	1.30 ± 0.03	1.29 ± 0.02	1.18 ± 0.02
LDL (mmol/L)	2.82 ± 0.06	2.88 ± 0.05	2.79 ± 0.06
Total cholesterol (mmol/L)	4.73 ± 0.09	4.79 ± 0.07	4.60 ± 0.10
Triglyceride (mmol/L)	1.72 ± 0.09	1.79 ± 0.07	2.23 ± 0.12
Lipoprotein (a) (g/L)	1.28 ± 0.02	1.26 ± 0.02	1.21 ± 0.02
Lipoprotein (b) (g/L)	0.98 ± 0.03	1.02 ± 0.02	1.04 ± 0.03
Blood glucose (mmol/L)	5.32 ± 0.15	5.33 ± 0.09	8.65 ± 0.29^∗^^#^
hs-TnI (ng/mL)	0.012 ± 0.003	0.253 ± 0.104^∗^	0.251 ± 0.006^∗^
CKMB (ng/mL)	1.01 ± 0.07	2.35 ± 0.64^∗^	2.97 ± 0.61^∗^

Data are presented as mean ± standard deviation or proportions. HDL: high-density lipoprotein; LDL: low-density lipoprotein; hs-TnI: high-sensitive troponin I; CKMB: creatine kinase MB. ^∗^*P* < 0.05 versus control; ^#^*P* < 0.05 versus CHD.

**Table 2:** Univariate regression analysis for the association of TMAO with demographic characteristics between CHD or CHD-T2DM patients and control participants.

Characteristic	CHD		CHD-T2DM	
	OR (95% CI)	*P* value	OR (95% CI)	*P* value
Age	1.066 (1.046–1.088)	0.110	1.083 (0.963–1.203)	0.124
Male	1.413 (0.911–2.190)	0.122	1.383 (1.160–1.606)	0.145
HDL	0.885 (0.436–1.795)	0.735	0.276 (0.119–0.635)	0.003
LDL	1.126 (0.827–1.534)	0.450	0.958 (0.705–1.302)	0.783
Total cholesterol	1.060 (0.867–1.295)	0.569	0.915 (0.747–1.120)	0.388
Triglyceride	1.073 (0.859–1.340)	0.534	1.366 (1.088–1.716)	0.007
Lipoprotein (a)	0.670 (0.263–1.709)	0.402	0.253 (0.084–0.764)	0.015
Lipoprotein (b)	1.438 (0.706–2.927)	0.317	1.456 (0.768–2.759)	0.249
Blood glucose	1.001 (0.865–1.158)	0.993	2.388 (1.874–3.043)	0.001
hs-TnI	1.737 (0.283–10.06)	0.371	1.724 (0.001–8.053)	0.013
CKMB	1.446 (1.044–2.002)	0.026	1.867 (1.305–2.672)	0.001
TMAO	3.621 (2.581–5.080)	0.001	9.581 (5.451–16.840)	0.001

**Table 3:** Multivariate regression analysis for the association of TMAO with demographic characteristics between CHD or CHD-T2DM patients and control participants.

Characteristic	CHD		CHD-T2DM	
	OR (95% CI)	*P* value	OR (95% CI)	*P* value
HDL	1.392 (0.218–8.868)	0.726	0.275 (0.011–6.876)	0.432
Triglyceride	1.144 (0.812–1.612)	0.434	1.196 (0.765–1.870)	0.434
Lipoprotein (a)	0.586 (0.045–7.657)	0.682	1.357 (1.001–2.134)	0.558
hs-TnI	2.599 (0.183–3.696)	0.481	1.290 (1.001–1.831)	0.285
CKMB	1.427 (0.994–2.048)	0.054	1.250 (0.742–2.106)	0.403
TMAO	3.469 (2.294–5.245)	0.001	9.110 (4.427–18.750)	0.001

**Table 4:** Correlation analysis for the association of TMAO with clinical parameters in CHD and CHD-T2DM patients.

Cardiovascular risk factors	TMAO			
	CHD		CHD-T2DM	
	Coefficient	*P* value	Coefficient	*P* value
HDL	0.174	0.001	0.167	0.006
LDL	0.009	0.865	0.006	0.920
Total cholesterol	0.145	0.006	0.018	0.759
Triglyceride	0.038	0.472	0.182	0.002
Lipoprotein (a)	0.034	0.525	0.109	0.072
Lipoprotein (b)	0.189	0.001	0.080	0.186
Blood glucose	0.031	0.564	0.176	0.003
hs-TnI	0.127	0.052	0.221	0.001
CKMB	0.125	0.034	0.216	0.001

## Data Availability

The data (determination of TMAO by LC-MS/MS) used to support the findings of this study are included within the article.
